# Radiomics Model for Predicting TP53 Status Using CT and Machine Learning Approach in Laryngeal Squamous Cell Carcinoma

**DOI:** 10.3389/fonc.2022.823428

**Published:** 2022-04-28

**Authors:** Ruxian Tian, Yumei Li, Chuanliang Jia, Yakui Mou, Haicheng Zhang, Xinxin Wu, Jingjing Li, Guohua Yu, Ning Mao, Xicheng Song

**Affiliations:** ^1^Department of Otorhinolaryngology, Head and Neck Surgery, The Affiliated Yantai Yuhuangding Hospital of Qingdao University, Yantai, China; ^2^Department of Radiology, Yantai Yuhuangding Hospital, Qingdao University, Yantai, China; ^3^Department of Pathology, Yantai Yuhuangding Hospital, Qingdao University, Yantai, China; ^4^Shandong Provincial Clinical Research Center for Otorhinolaryngologic Diseases, Yantai, China

**Keywords:** TP53, laryngeal squamous cell carcinoma, computed tomography, radiomics, machine learning

## Abstract

**Objective:**

We aim to establish and validate computed tomography (CT)-based radiomics model for predicting TP53 status in patients with laryngeal squamous cell carcinoma (LSCC).

**Methods:**

We divided all patients into a training set 1 (n=66) and a testing set 1 (n=30) to establish and validate radiomics model to predict TP53. Radiomics features were selected by analysis of variance (ANOVA) and the least absolute shrinkage and selection operator (Lasso) regression analysis. Five radiomics models were established by using K-Nearest Neighbor, logistics regressive, linear-support vector machine (SVM), gaussian-SVM, and polynomial-SVM in training set 1. We also divided all patients into a training set 2 and a testing set 2 according to different CT equipment to establish and evaluate the stability of the radiomics models.

**Results:**

After ANOVA and subsequent Lasso regression analysis, 22 radiomics features were selected to build the radiomics model in training set 1. The radiomics model based on linear-SVM has the best predictive performance of the five models, and the area under the receiver operating characteristic curve in training set 1 and testing set 1 were 0.831(95% confidence interval [CI] 0.692–0.970) and 0.797(95% CI 0.632–0.957) respectively. The specificity, sensitivity, and accuracy were 0.971(95% CI 0.834–0.999), 0.714(95% CI 0.535–0.848), and 0.843(95% CI 0.657–0.928) in training set 1 and 0.750(95% CI 0.500–0.938), 0.786(95% CI 0.571–1.000), and 0.667(95% CI 0.467–0.720) in testing set 1, respectively. In addition, the radiomics model also achieved stable prediction results even in different CT equipment. Decision curve analysis showed that the radiomics model for predicting TP53 status could benefit LSCC patients.

**Conclusion:**

We developed and validated a relatively optimal radiomics model for TP53 status prediction by trying five different machine learning methods in patients with LSCC. It shown great potential of radiomics features for predicting TP53 status preoperatively and guiding clinical treatment.

## Introduction

Laryngeal squamous cell carcinoma (LSCC) represents one-third of all head and neck squamous cancer (HNSCC); about 60% of the patients were discovered and diagnosed at its advanced stage (1, 2). Despite advances in radiotherapy, chemotherapy, and surgery for LSCC over the past decades, the 5-year survival rate for laryngeal cancer has not significantly improved (3, 4). Currently, clinicians formulate treatment strategies for LSCC based on TNM stage and primary site. However, due to heterogeneity in the biological and molecular pathogenesis of HNSCC, even tumors at the same TNM stage may respond differently to the same therapy. Therefore, identifying applicable biomarkers is necessary for predicting prognosis and stratifying patients to develop individualized therapeutic plans.

TP53 was a tumor suppressor gene; its mutation was often detected in LSCC and is often involved in tumorigenesis and development (5). Studies have indicated that compared with wild-type TP53, mutant TP53 could promote tumor cell proliferation and metastasis (6). Several reports have shown that TP53 mutation was related to shortened survival time and resistance to therapy in patients with LSCC (7). As a result, TP53 mutation status could be considered as a biomarker for risk stratification and prediction of clinical treatment response in patients with LSCC. Clinically, TP53 mutation status was determined by DNA sequencing analysis of excised tissues, but this method cannot be applied to non-surgical patients. In addition, this was an invasive detection method with high cost, and more importantly, the tissue sample used for the detection may not accurately reflect the intratumorally heterogeneity. Thus, a non-invasive and low-cost method that could reflect intratumorally heterogeneity is needed to help identify TP53 status in LSCC patients.

As an emerging field, radiomics refers to the conversion of a large number of medical images into high-dimensional, mineable, and quantitative imaging features *via* high-throughput extraction of data-characterization algorithms for clinical decision (8). In recent years, the application of radiomics to the prediction of genotype, lymph node metastasis, survival prognosis, and evaluation of treatment response has been the focus of researchers (9–12). Radiogenomics analysis showed that the imaging features could reflect the intratumorally heterogeneity and were associated with potential gene expression patterns (13). Huang et al. showed that radiomics has the potential to identify treatment-related gene subtypes of head and neck squamous cell carcinoma, which provides the possibility for patient stratification and precise treatment (14). In addition, a series of pioneer studies have shown the promise of computed tomography (CT) radiomics features for predicting gene status (15–18).

The status of medical imaging technology in clinical oncology has been continuously improved (19). As a routine examination for laryngeal cancer patients, contrast-enhanced CT was a non-invasive examination method, whose images were easy to acquire and could reflect the intratumorally heterogeneity of the entire tumor. To our knowledge, no specific studies have been reported on the relationship between CT features and TP53 status in LSCC. Therefore, we developed five CT-based radiomics model by trying different machine learning approaches to predict TP53 status in patients with LSCC and providing preliminary performance testing.

## Materials and Methods

### Patients

**Figure 1:**
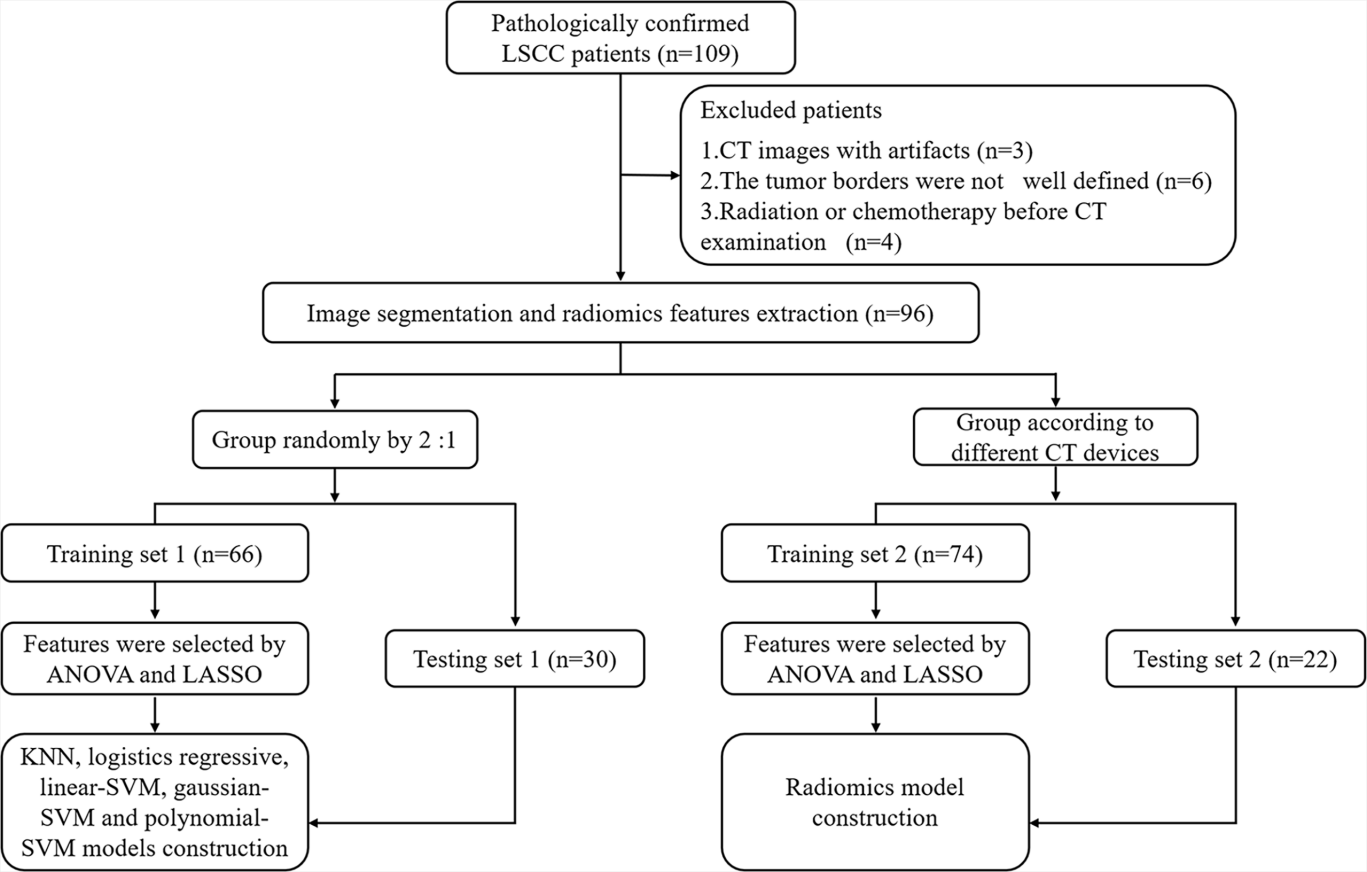
Radiomics workflow and study flowchart. LSCC, laryngeal squamous cell carcinoma; ANOVA, analysis of variance; LASSO, least absolute shrinkage and selection operator; KNN, K-Nearest Neighbor; SVM, support vector machine.

A total of 96 patients (93 men and 3 women; mean age, 62.13 ± 8.96 years) with LSCC, who were treated at the Yuhuangding Hospital from January 2016 to May 2020, were included in this retrospective study. The inclusion criteria were as follows: 1. pathological examination confirmed LSCC; 2. contrast-enhanced CT examination within 2 weeks before operation; 3. TP53 status was determined by DNA sequencing analysis; and 4. available clinical characteristics. The exclusion criteria were showed in **Figure 1**.

We divided all patients into a training set 1 and a testing set 1 at a ratio of approximately 2:1 to establish and validate radiomics model for predicting TP53. In addition, to verify the impact of different CT equipment on the prediction results, we selected 74 patients examined by a CT scanner (PHILIPS Brilliance 64) as the training set 2 and 22 patients examined by other CT scanners (SIEMENS Sensation 64; GE Light Speed VCT XT64) as the testing set 2. Clinical characteristics included age, gender, T stage, N stage, TNM stage, smoking history, drinking history, tumor location, histological grade, and family history of malignancy. This retrospective study was approved by the Institutional Review Board and all participants signed an informed consent.

### TP53 Mutation Detection

All surgical tissue specimens were fixed with formaldehyde and embedded with paraffin. Three to five sections were taken to scrape the tissue in the rich region of tumor cells, as compared with the HE sections. DNA extraction was conducted in accordance with the instructions (Amoydx ^®^ FFPE DNA Kit, Amoy Diagnostics, Xiamen, China). TP53 status was evaluated by PCR amplification and DNA sequencing. The reaction system of PCR amplification, including target DNA 1 μL, 2×PCR reaction buffer 12.5 μL, each primer (10 μM) 2 μL, and ddH_2_O, was supplemented at 25 μL. Cyclic parameters were as follows: pre-denaturation at 95°C for 5 min; 95°C for 30 s, 55°C for 30 s, 72°C for 1 min, for a total of 31 cycles; and extension at 72°C for 5 min. The amplified product was electrophoresis in a 1.5% agarose gel. The amplified products were recovered by gel-cutting and identified by agarose gel electrophoresis. The bidirectional sequencing analysis was conducted by Sanger sequencing method with ABI3500DX gene sequencer. This method was currently the accepted standard for identifying TP53 mutations.

### Image Acquisition and Segmentation

All patients underwent contrast-enhanced larynx CT with a 64-slice spiral CT scanner, scanning range from the upper mediastinum to the skull base. After plain CT scanning, a dynamic contrast-enhanced CT scan was performed after intravenous administration of 80–100 ml nonionic contrast material (Iopamidol, 370 mg I/ml, Bracco, Milan, Italy) using power injection at a rate of 3.5 ml/s followed by saline flush (20 ml). Arterial phase and venous phase images were obtained at 30 and 65 s, respectively. The slice thickness of the reconstructed image was 1.0 mm. Arterial phase, venous phase, and plain scan CT images were retrieved for image feature extraction.

**Figure 2:**
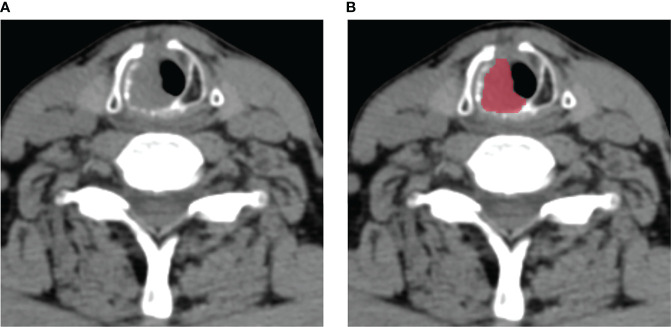
An example of manual segmentation in laryngeal squamous cell carcinoma (LSCC). **(A)** Localized space-occupying lesion of LSCC was observed on plain CT image; **(B)** Manual segmentation on the same axial slice was depicted with red label.

The radiomics workflow was presented in **Figure 1**. All the images were uploaded to Huiyihuiying platform (www.huiyihuiying.com) for manual segmentation to obtain the region of interest (ROI) for subsequent radiomics feature extraction. The ROI of each patient obtained manually depicted the outline of the tumor, which was then integrated into a volume of interest (VOI). The details are shown in **Figure 2**. The manual segmentation process was done by radiologist 1 with 10 years of experience. Two months later, radiologist 1 and radiologist 2 with 10 years of experience randomly selected 40 patients in the training set 1 and segmented their images again to assess the intra-/inter-reader agreement of the radiomics analysis. The intraobserver intraclass correlation coefficient (ICC) was used to assess the agreement of radiomics features extracted by radiologist 1. The interobserver ICC was used to assess the agreement of radiomics features extracted by the two radiologists. ICCs > 0.8 has good consistency.

### Radiomics Features Extraction

PyRadiomics (https://pyradiomics.readthedocs.io/en/latest/index.html) was an open-source package that was recommended for standardized radiomics analysis and extraction of radiomics features. The radiomics features we extracted include first-order statistics, shape-based features, and texture features. To reduce the bias caused by different acquisition parameters, all imaging data were normalized (Z-value transformation) before feature extraction. After assessing the consistency, we respectively extracted 1409 robust radiomics features, with ICCs of > 0.8, from the plain scan, arterial, and venous phases of each patient in the training set. Finally, a total of 1409×3 radiomics features were extracted for each patient in the training set.

### Feature Selection and Radiomics Model Building

Radiomics features were preliminarily screened by analysis of variance (ANOVA), and features with *P* < 0.05 were associated with TP53 mutation status in the training set. The least absolute shrinkage and selection operator (Lasso) regression was used to further select the optimal radiomics features from the training set. The model used tuning parameters (alpha) to select features. The coefficients of some covariates may shrink toward zero as they become smaller. Then, we chose alpha when the cross-testing error was smallest. This method reduced most of the coefficients to zero, and the remaining non-zero coefficients are selected by Lasso. To avoid over-fitting, 5-fold cross testing was adopted.

We respectively used five machine learning approach, namely, K-Nearest Neighbor (KNN), logistics regressive, linear- support vector machine (SVM), Gaussian-SVM, and polynomial-SVM, to build the radiomics model for predicting TP53 status based on the features selected by Lasso in the training set 1. In addition, we chose one of the five machine learning methods with better performance to establish a radiomics model in training set 2 and testing set 2 to verify the impact of different CT equipment on the prediction results.

### Predictive Performance of the Radiomics Model

The predictive performance of the radiomics models was evaluated through the receiver operating characteristic (ROC) curve and area under the curve (AUC) in the training set, and verified in the testing set. Specificity, sensitivity, and accuracy were also used to evaluate the predictive performance of the models. Decision curve analysis (DCA) was applied to evaluate the clinical utility of the five models by calculating the net benefits within the threshold probability range.

### Statistical Analysis

Clinical data were analyzed using R software (version 4.0.4, TUNA Team, Tsinghua University, https://mirrors.tuna.tsinghua.edu.cn/CRAN/). Non-parametric quantitative data were presented as a median value and interquartile range [*M*(*P_25_*-*P_75_*)], and Mann-Whitney U test was used to analyze age differences between patients. Chi-square test was used to analysis the differences between classified data. The selection of clinical data and DCA was conducted based on “rms” and “rmda” packages. The third party module sklearn (http://scikit-learn.org/stable/index.html) of Python software (version 3.6.1 for windows) was used for feature selection and machine learning model construction. A two-sided *P* < 0.05 was considered to be statistically significant.

## Results

### Clinical Characteristics

A total of 45 (46.88%) of the 96 patients showed TP53 mutation, including 31 (46.97%) in the training set 1 and 14 (46.67%) in the testing set 1. The TP53 mutation information of patients with laryngeal cancer is shown in **Supplementary Table S1**. No statistically significant difference in the proportion of TP53 mutation was found between the training set 1 and testing set 1 (*P* = 0.978). No statistically significant differences were found in clinical characteristics between the mutated and wild-type groups in either the training set 1 and testing set 1 (all *P* values > 0.05, **Table 1**) or the training set 2 and testing set 2 (all *P* values > 0.05, **Supplementary Table S2**). **Supplementary Table S1**

**Table 1:** Clinical characteristics of patients in training set 1 and testing set 1 in the wild-type group and mutated group.

Characteristics	Training set 1		*P*	Testing set 1		*P*
	Wild-type group	Mutated group		Wild-type group	Mutated group	
Age (Mean ± SD, years)	61.78 ± 3.02	62.93 ± 5.07	0.348	62.24 ± 3.43	60.28 ± 4.73	0.176
Gender, *n* (%)			0.494			0.467
Male	33 (94.29)	31 (100)		16 (100)	13 (92.86)	
female	2 (5.71)	0 (0)		0 (0)	1 (7.14)	
Tumor location, *n* (%)			0.563			0.972
Supraglottis	5 (14.29)	6 (19.35)		4 (25.00)	3 (21.43)	
Glottis	29 (82.86)	25 (80.65)		11 (68.75)	10 (71.43)	
Subglottis	1 (2.85)	0 (0)		1 (6.25)	1 (7.14)	
T stage, *n* (%)			0.132			0.296
T1	6 (17.14)	7 (22.58)		5 (31.25)	1 (7.14)	
T2	16 (45.71)	6 (19.35)		5 (20.00)	5 (35.71)	
T3	8 (22.86)	13 (41.94)		4 (25.00)	7 (50.00)	
T4	5 (14.29)	5 (16.13)		2 (12.50)	1 (7.14)	
N stage, *n* (%)			0.094			0.151
N0	28 (80.00)	19 (61.29)		13(81.25)	8 (57.14)	
N1, N2	7 (20.00)	12 (38.71)		3(18.75)	6 (42.86)	
TNM stage, *n* (%)			0.099			0.424
I	6 (17.14)	7 (22.58)		5 (31.25)	1 (7.14)	
II	14 (40.00)	4 (12.90)		4 (25.00)	4 (28.57)	
III	8 (22.86)	12 (38.71)		4 (25.00)	5 (35.71)	
IV	7 (20.00)	8 (25.81)		3 (18.75)	4 (28.57)	
Histologic grade, *n* (%)			0.064			0.503
Poor	5 (14.29)	9 (29.03)		3 (18.8)	3 (21.43)	
Moderate	20 (57.14)	9 (29.03)		10 (62.5)	6 (42.86)	
Well	10 (28.57)	13 (41.94)		3 (18.8)	5 (35.71)	
Smoking, *n* (%)			1.000			0.586
Yes	30 (85.71)	27 (87.10)		15 (93.75)	12 (85.71)	
No	5(14.29)	4 (12.90)		1 (6.25)	2 (14.29)	
Drinking, *n* (%)			0.792			0.675
Yes	23 (65.71)	22 (70.97)		13 (81.25)	10 (71.4)	
No	12 (34.29)	9 (29.03)		3 (18.75)	4 (28.6)	
Family history of cancer, *n* (%)			0.265			0.157
Yes	6 (17.14)	2 (6.45)		1 (6.25)	4 (28.57)	
No	29 (82.86)	29 (93.55)		15 (93.75)	10 (71.43)	

### Feature Selection and Radiomics Model Building

**Figure 3:**
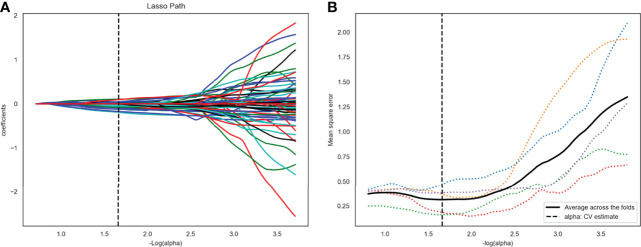
Twenty-two radiomics features were selected using the least absolute shrinkage and selection operator algorithm (LASSO). **(A)** The LASSO coefficient profiles of the 117 radiomic features. Each colored line represents a coefficient corresponding to each feature. A vertical line is drawn at the value where the optimal alpha results in 22 nonzero coefficients. **(B)** Mean square error path using five-fold cross-testing.

After ANOVA, 117 of 1409 radiomics features related to TP53 status were screened out from the plain scan phase in the training set 1. No correlation was found between TP53 status and radiomics features from other CT scan phases. Subsequently, 22 potential features that could predict TP53 mutation were selected by Lasso regression analysis in the training set 1 (**Figure 3**). The 22 detailed radiomics features were listed in **Supplementary Table S3**. In the same way, 107 of 1409 radiomics features related to TP53 status were screened out in the training set 2, and 17 features remained after Lasso regression analysis (**Supplementary Figure S1** and **Table S4**).

Multivariate analysis shown no association between clinical characteristics and TP53 status. So, five radiomics models for predicting TP53 status were established in the training set 1 by using the selected 22 features and KNN, logistic regressive, linear-SVM, Gaussian-SVM, and polynomial-SVM.

### Predictive Performance of Radiomics Model

**Figure 4:**
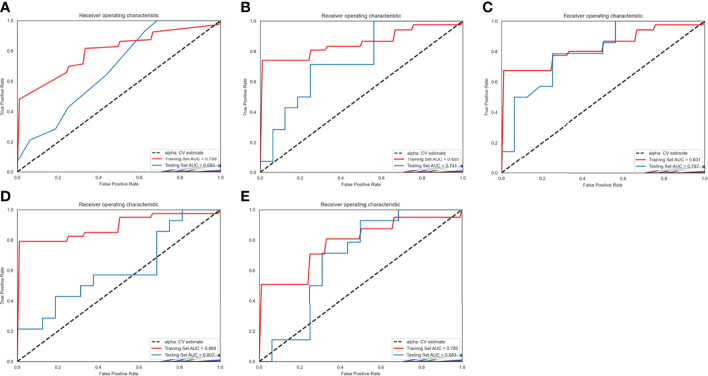
Receiver operating characteristic (ROC) curves of radiomics models based on different machine learning methods in training set 1 and testing set 1. **(A–E)** ROC curves for the model based on K-Nearest Neighbor, logistic regressive, linear-support vector machine (SVM), Gaussian-SVM, and polynomial-SVM, respectively.

**Figure 5:**
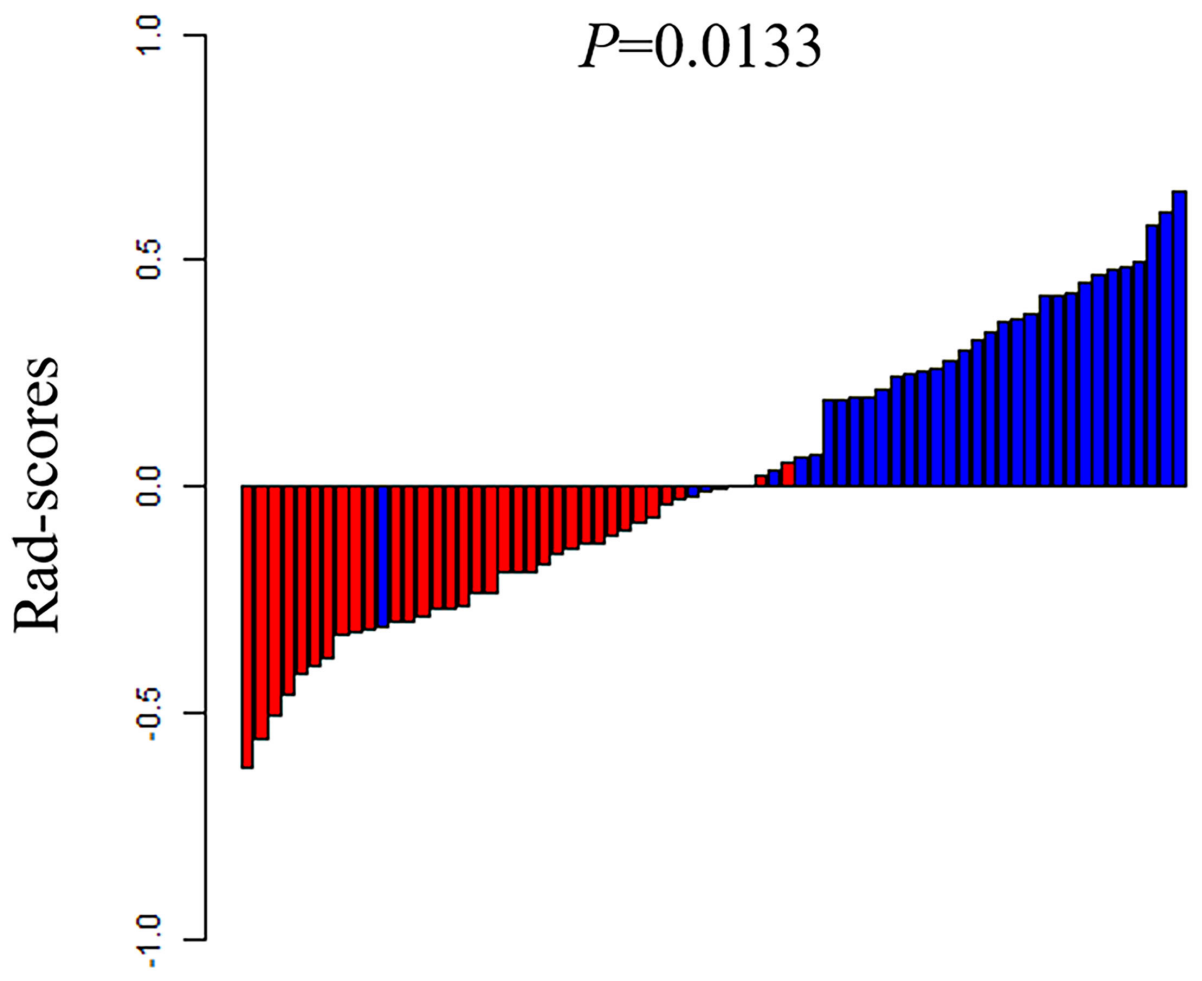
The rad-scores of each patient in the training set 1. Red represents the TP53 wild type and blue represents the mutated TP53.

The ROC of the five radiomics models in training set 1 and testing set 1 are shown in **Figure 4**. The AUC, specificity, sensitivity, and accuracy of the five radiomics models in training set 1 and testing set 1 are shown in **Tables 2** and **3**. The radiomics model based on linear-SVM had the best performance among the above mentioned models. The AUC, specificity, sensitivity, and accuracy in training set 1 were 0.831(95% confidence interval [CI] 0.712–0.930), 0.971(95% CI 0.834–0.999), 0.714(95% CI 0.535–0.848), and 0.843(95% CI 0.647–0.942), respectively. In the testing set 1, the AUC, specificity, sensitivity, and accuracy of the radiomics model were 0.797(95% CI 0.632–0.957), 0.750(95% CI 0.500–0.938), 0.786(95% CI 0.571–1.000), and 0.667(95% CI 0.472–0.827), respectively. Taking the linear-SVM classifier as an example, the radiomics scores of the mutated group was significantly higher than that of the wild-type group in training set 1 (**Figure 5**).

**Table 2:** Performance of the different radiomics models in the training set 1.

	AUC (95%CI)	Specificity (95%CI)	Sensitivity (95%CI)	Accuracy (95%CI)
KNN	0.799 (0.625-0.909)	0.657 (0.477-0.803)	0.829 (0.657-0.928)	0.743 (0.570-0.879)
Logistics Regression	0.855 (0.735-0.956)	0.971 (0.834-0.999)	0.800 (0.625-0.909)	0.857 (0.675-0.930)
Linear-SVM	0.831 (0.712-0.930)	0.971 (0.834-0.999)	0.714 (0.535-0.848)	0.843 (0.647-0.942)
Gaussian-SVM	0.888 (0.750-0.963)	0.971 (0.834-0.999)	0.793 (0.597-0.913)	0.814 (0.634-0.912)
Polynomial-SVM	0.782 (0.612-0.892)	0.829 (0.657-0.928)	0.657 (0.477-0.803)	0.743 (0.570-0.879)

CI, confidence interval; AUC, area under ROC curve; KNN, K-Nearest Neighbor; SVM, support vector machine.

**Table 3:** Performance of the different radiomics models in the testing set 1.

	AUC (95%CI)	Specificity (95%CI)	Sensitivity (95%CI)	Accuracy (95%CI)
KNN	0.683 (0.472-0.768)	0.333 (0.113-0.646)	1.000 (0.781-1.000)	0.600 (0.406-0.773)
Logistics Regression	0.741 (0.569-0.856)	0.750 (0.474-0.917)	0.714 (0.420-0.904)	0.733 (0.541-0.877)
Linear-SVM	0.797 (0.632-0.957)	0.750 (0.500-0.938)	0.786 (0.571-1.000)	0.667 (0.472-0.827)
Gaussian-SVM	0.607 (0.467-0.720)	0.286 (0.096-0.580)	0.875 (0.604-0.978)	0.600 (0.406-0.773)
Polynomial-SVM	0.683 (0.506-0.826)	0.500 (0.255-0.745)	0.929 (0.642-0.996)	0.700 (0.504-0.848)

CI, confidence interval; AUC, area under ROC curve; KNN, K-Nearest Neighbor; SVM, support vector machine.

In addition, we chose linear-SVM to build the radiomics model in training set 2. The ROC of the radiomics model established was shown in **Supplementary Figure S2**. The AUC, specificity, sensitivity, and accuracy of the radiomics model in training set 2 were 0.877(95% CI 0.814–0.933), 0.822(95% CI 0.674–0.915), 0.795(95% CI 0.631–0.901), and 0.810(95% CI 0.709–0.887) respectively. In testing set 2, the AUC, specificity, sensitivity, and accuracy of the radiomics model were 0.750(95% CI 0.576–0.882), 0.667(95% CI 0.241–0.904), 0.833(95% CI 0.365–0.991), and 0.750(95% CI 0.576–0.882), respectively.

**Figure 6:**
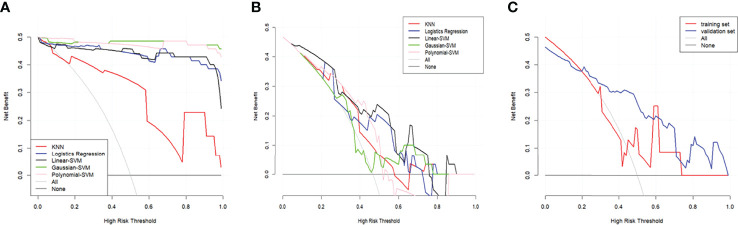
Decision curve analysis (DCA) for the radiomics models. **(A)** DCA for the five radiomics models in training set 1. **(B)** DCA for the five radiomics models in testing set 1. **(C)** DCA for the radiomics model based on liner-SVM in training set 2 and testing set 2. The gray line is the decision curve for treat all patients assuming TP53 status as mutation. The black line is the decision curve for treat none patients which assumes TP53 status as wild-type.

The DCA of the five radiomics models in training set 1 and testing set 1 was shown in **Figures 6A, B**. All of the models were better than treating all patients or not treating all patients under the corresponding threshold probability. The DCA of the radiomics model based on linear-SVM in training set 2 showed the same trend in **Figure 6C**.

## Discussion

Our study established five CT-based radiomics models by using different machine learning approach for predicting the status of TP53 in LSCC. The five prediction models could identify the status of TP53 in LSCC and showed good prediction performance both in the training and testing sets, and even in different examination equipment. CT radiomics features were helpful for identifying TP53 status and thus had the potential to provide assistance for the clinical management of patients with LSCC.

With the development of radiomics, the relationship between radiomics features and gene status was gradually revealed. Regarding TP53, a PET/CT-based radiomics study found an increased value of short-run low gray-level emphasis derived from the gray-level run length matrix in patients with TP53 mutation (20). Another previous study in lung cancer showed a strong correlation between CT imaging features and TP53 mutation status (21). In our study, the TP53 status was closely related to the radiomics features extracted from CT of laryngeal cancer, and the prediction model based on the selected features could effectively predict TP53 status. These findings confirmed a wide association between TP53 mutation status and radiomics features in tumors. In addition, Li et al. established a machine learning model to predict TP53 mutations in gliomas by using SVM and imaging features, and the model had a good discriminative performance in both the training set and the testing set (6). Their study also found that clinical features were not associated with TP53 mutations, which was consistent with our findings. However, one thing that was obviously different from our model was the features they extracted from MRI. Both studies showed the feasibility of machine learning-based radiomics model for predicting TP53 status in tumors.

In HNSCC, the relevant studies also reported that the CT-based radiomics model could predict TP53 mutation status. Zhu et al.’s research on HNSCC showed that gene features were widely related to imaging features reflecting tumor size, shape, and texture (22). Furthermore, they used random forest classifier to develop a radiomics model that could predict TP53 mutation status, and the AUC of its model was 0.641. Huang et al. developed and validated a radiomics model for predicting the molecular characteristics and subtypes of head and neck tumors, which had an AUC of 0.650 when predicting TP53 mutation status (14). The above two studies used only one algorithm to establish a prediction model, while our study used five different machine learning methods to predict TP53 status. Our study could better reflect the feasibility and applicability of CT features in predicting gene status. In addition, the AUC of the radiomics model in these two studies for predicting TP53 status was lower than that in our study. As we know, HNSCC includes a variety of tumors that may originate in the pharynx, larynx, and oral cavity. Ledgerwood et al. demonstrated differences in mutational heterogeneity by different subsites in HNSCC, which may result in a lower predictive performance than the radiomics model in LSCC alone (23). In addition, different feature selection and modelling methods may also lead to a difference in the predictive performance of the radiomics model. Another notable advantage of our study was that the model was also established according to different examination equipment and showed good prediction performance in the testing set, which ruled out the possible influence of examination equipment on the prediction results, and further demonstrated the applicability of CT features in predicting TP53 status.

To obtain the optimal predictors in the process of building the radiomics model, 122 CT features were reduced to 22 by Lasso algorithm. Lasso was often used in the process of establishing predictive models in radiomics, because it was particularly suitable for selecting variables from high-throughput data and could avoid overfitting (24, 25). At present, the radiomics model established by machine learning method has shown good performance in predicting disease diagnosis, survival prognosis, pathological grading, and gene status (26–29). In our study, five machine learning models were established based on the features selected by Lasso; all these models showed good performance in distinguishing TP53 status in LSCC patients.

This study still has some shortcomings. First of all, because this study was a retrospective design, all the image data came from a single center, and the sample size was small. Thus, potential selection bias cannot be ruled out, which inevitably reduced the reliability of the radiomics model. However, our study confirmed the potential of CT-based radiomics in predicting the status of TP53 in laryngeal cancer. Therefore, multi-center and large-sample prospective studies are needed to improve the predictive performance of the radiomics model. Second, despite the use of ICCs to select the robust features, there may still be feature variations in manual segmented images. Third, further radiogenomics analysis and experiments are needed to explain the pathophysiological process behind the radiomics features of each included model.

Our study found an association between radiomics features and TP53 mutations in LSCC. By trying five different machine learning methods, we finally established a radiomics model that best predicted the TP53 status of patients with LSCC and demonstrated the ability to predict TP53 status noninvasively and efficiently in patients with LSCC.

## Data Availability Statement

The raw data supporting the conclusions of this article will be made available by the authors, without undue reservation.

## Ethics Statement

This retrospective study was approved by the Institutional Review Board and all participants signed an informed consent. The patients/participants provided their written informed consent to participate in this study.

## Author Contributions

RT and YL contributed to the data analysis and the manuscript preparation. XS, NM, and RT contributed to the conception and design of the study. CJ, HZ, JL, and XW contributed to data acquisition. GY is responsible for pathological analysis. XS, NM, and YM contributed to the manuscript revision. All authors contributed to the article and approved the submitted version.

## Funding

This work was supported by Taishan Scholars Project (No. ts20190991).

## Conflict of Interest

The authors declare that the research was conducted in the absence of any commercial or financial relationships that could be construed as a potential conflict of interest.

## Publisher’s Note

All claims expressed in this article are solely those of the authors and do not necessarily represent those of their affiliated organizations, or those of the publisher, the editors and the reviewers. Any product that may be evaluated in this article, or claim that may be made by its manufacturer, is not guaranteed or endorsed by the publisher.
