# Supplementary material for: Radiomics Model for Predicting TP53 Status Using CT and Machine Learning Approach in Laryngeal Squamous Cell Carcinoma
Source: Front Oncol. 2022 Apr 28;12:823428. doi: 10.3389/fonc.2022.823428 (PMC9095903; doi:10.3389/fonc.2022.823428)
Supplement: Supplementary file 1 [file DataSheet_1.docx]

Supplementary Material

1. Supplementary Tables

| **Table S1** TP53 mutation information in patients with laryngeal cancer (*n*) | | | |
| --- | --- | --- | --- |
|  | Exon 5 | Exon 7 | Exon 8 |
| Training set 1 | 18 | 8 | 5 |
| Testing set 1 | 8 | 4 | 2 |

| **Table S2** Clinical characteristics of patients in training set 2 and testing set 2 in the wild-type group and mutated group | | | | | | |
| --- | --- | --- | --- | --- | --- | --- |
| Characteristics | Training set 2 74 | | *P* | Testing set 2 22 | | *P* |
|  | Wild-type group | Mutated group |  | Wild-type group | Mutated group |  |
| Age (Mean ± SD, years) | 62.00±9.17 | 63.45±9.18 | 0.523 | 60.75±8.54 | 61.00±7.45 | 0.935 |
| Gender, *n* (%) |  |  | 0.654 |  |  | / |
| Male | 38(95.00) | 33(97.06) |  | 12(100) | 10(100) |  |
| female | 2(5.00) | 1(2.94) |  | 0(0) | 0(0) |  |
| Tumor location, *n* (%) |  |  | 0.993 |  |  | 0.528 |
| Supraglottis | 7(17.50) | 6(17.65) |  | 2(16.67) | 3(30.00) |  |
| Glottis | 32(80.00) | 27(79.41) |  | 9(75.00) | 7(70.00) |  |
| Subglottis | 1(2.50) | 1(2.94) |  | 1(8.33) | 0(0) |  |
| T stage, *n* (%) |  |  | 0.548 |  |  | 0.599 |
| T1 | 7(17.50) | 9(26.47) |  | 2(16.67) | 1(10.00) |  |
| T2 | 16(40.00) | 9(26.47) |  | 3(25.00) | 4(40.00) |  |
| T3 | 10(25.00) | 11(32.35) |  | 6(50.00) | 5(50.00) |  |
| T4 | 7(17.50) | 5(14.71) |  | 1(8.33) | 0(0) |  |
| N stage, *n* (%) |  |  | 0.649 |  |  | 0.484 |
| N0 | 29(72.50) | 23(67.65) |  | 8(66.67) | 8(80.00) |  |
| N1, N2 | 11(27.50) | 11(32.35) |  | 4(33.33) | 2(20.00) |  |
| TNM stage, *n* (%) |  |  | 0.524 |  |  | 0.648 |
| Ⅰ | 10(25.00) | 7(20.59) |  | 1(8.33) | 1(10.00) |  |
| Ⅱ | 10(25.00) | 5(14.71) |  | 7(58.33) | 4(40.00) |  |
| Ⅲ | 12(30.00) | 11(32.35) |  | 2(16.67) | 4(40.00) |  |
| Ⅳ | 8(20.00) | 11(32.35) |  | 2(16.67) | 1(10.00) |  |
| Histologic grade, *n* (%) |  |  | 0.992 |  |  | 0.725 |
| Poor | 9(22.50) | 8(23.53) |  | 2(16.67) | 1(10.00) |  |
| Moderate | 17(42.50) | 14(41.18) |  | 8(66.67) | 6(60.00) |  |
| Well | 14(35.00) | 12(35.29) |  | 2(16.67) | 3(30.00) |  |
| Smoking, *n* (%) |  |  | 0.972 |  |  | 0.262 |
| Yes | 34(85.00) | 29(85.29) |  | 12(100) | 9(90.00) |  |
| No | 6(15.00) | 5(14.71) |  | 0(0) | 1(10.00) |  |
| Drinking, *n* (%) |  |  | 0.800 |  |  | 0.427 |
| Yes | 27(67.50) | 22(64.71) |  | 11(91.67) | 8(80.00) |  |
| No | 13(32.50) | 12(35.29) |  | 1(8.33) | 2(20.00) |  |
| Family history of cancer, *n* (%) |  |  | 0.685 |  |  | 0.650 |
| Yes | 6(15.00) | 4(11.76) |  | 2(16.67) | 1(10.00) |  |
| No | 34(85.00) | 30 (88.24) |  | 10(83.33) | 9(90.00) |  |

| **Table S3** Twenty-two radiomics features selected by the LASSO in trianing set 1 | |
| --- | --- |
| Radiomics features | coefficient |
| wavelet-HLH_glszm_GrayLevelNonUniformityNormalized_p | 0.046085628 |
| exponential_firstorder_90Percentile_p | 0.097964733 |
| wavelet-HLH_firstorder_Kurtosis_VP | -0.17107969 |
| wavelet-LLH_firstorder_Median_p | 0.015348994 |
| wavelet-LLH_glrlm_LowGrayLevelRunEmphasis_p | -0.110087642 |
| wavelet-LLL_firstorder_InterquartileRange_VP | -0.148711248 |
| wavelet-LHH_firstorder_Skewness_VP | 0.002487449 |
| wavelet-HHH_glrlm_GrayLevelNonUniformityNormalized_p | -0.082619201 |
| wavelet-HHH_glrlm_GrayLevelVariance_p | 2.42E-07 |
| original_glszm_ZoneVariance_p | 0.05156866 |
| wavelet-LLL_firstorder_Range_p | 0.046853033 |
| wavelet-HLH_glszm_GrayLevelNonUniformity_p | 0.039637204 |
| wavelet-HHH_firstorder_Mean_p | -0.068844533 |
| wavelet-HHH_glszm_SmallAreaEmphasis_p | -0.006239706 |
| logarithm_firstorder_Kurtosis_p | -0.030785772 |
| wavelet-LHH_firstorder_Kurtosis_p | 0.08410243 |
| wavelet-HLL_firstorder_Skewness_p | -0.022899742 |
| wavelet-LLH_glszm_SmallAreaEmphasis_p | -0.018995057 |
| wavelet-HLH_glszm_LargeAreaHighGrayLevelEmphasis_p | -0.085943361 |
| wavelet-LLH_firstorder_Kurtosis_p | -0.054717551 |
| wavelet-LHL_glrlm_RunVariance_VP | -0.070212102 |
| wavelet-HHH_glcm_SumEntropy_p | -0.042681599 |

| **Table S4** Seventeen radiomics features selected by the LASSO in trianing set 2 | |
| --- | --- |
| Radiomics features | coefficient |
| wavelet-HHH_glszm_SmallAreaLowGrayLevelEmphasis_p | -0.071927976 |
| wavelet-HLH_glszm_GrayLevelNonUniformityNormalized_p | 0.083405891 |
| wavelet-HHL_glcm_Imc1_p | -0.003074739 |
| wavelet-HHL_glcm_MCC_p | 0.044663282 |
| original_shape_Maximum2DDiameterSlice_p | -0.031784829 |
| wavelet-LLH_glszm_SmallAreaHighGrayLevelEmphasis_p | -0.011185935 |
| wavelet-HHL_firstorder_Kurtosis_p | -0.032320494 |
| wavelet-HLH_glszm_SizeZoneNonUniformity_p | -0.012046203 |
| logarithm_firstorder_Range_AP | -0.009245612 |
| wavelet-HLH_gldm_DependenceVariance_p | 0.020055471 |
| wavelet-LLH_glcm_ClusterShade_p | -0.021230992 |
| wavelet-HHL_glszm_SizeZoneNonUniformity_p | -0.030050373 |
| wavelet-HHL_glcm_Idn_p | -0.062832402 |
| wavelet-HHL_glcm_Contrast_p | 3.05E-08 |
| wavelet-HHL_glcm_Idmn_p | -8.99E-09 |
| original_firstorder_90Percentile_p | 0.024548683 |
| wavelet-LLH_gldm_HighGrayLevelEmphasis_p | 0.011795458 |

**2. Supplementary Figures**


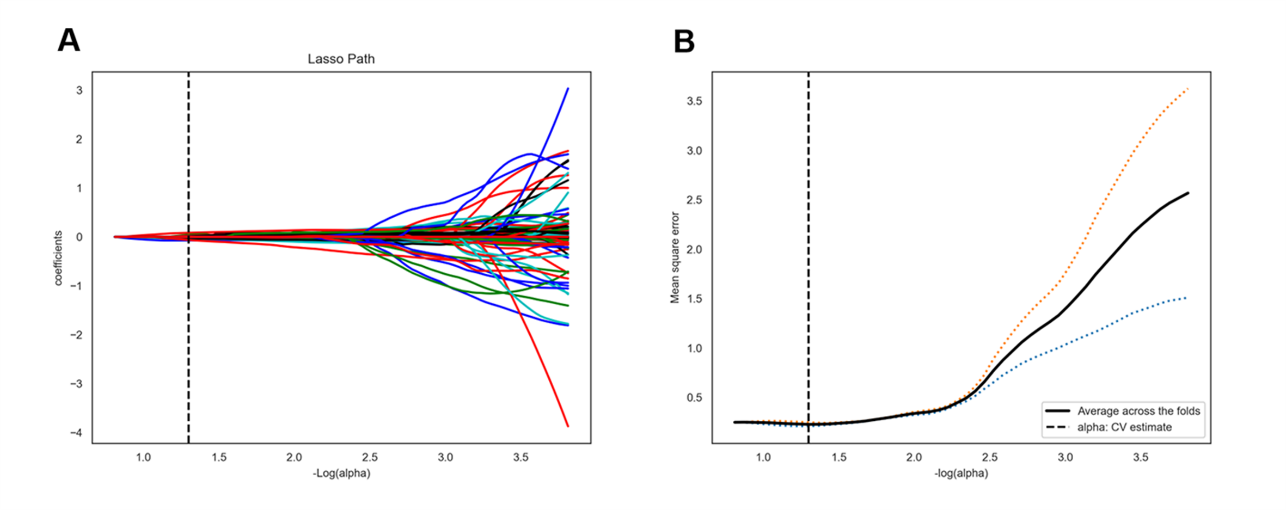


**Supplementary Figure 1.** Seventeen radiomics features were selected using the least absolute shrinkage and selection operator algorithm (LASSO). (A) The LASSO coefficient profiles of the 107 radiomic features. Each colored line represents a coefficient corresponding to each feature. A vertical line is drawn at the value where the optimal alpha results in 17 nonzero coefficients. (B) Mean square error path using five-fold cross-testing.


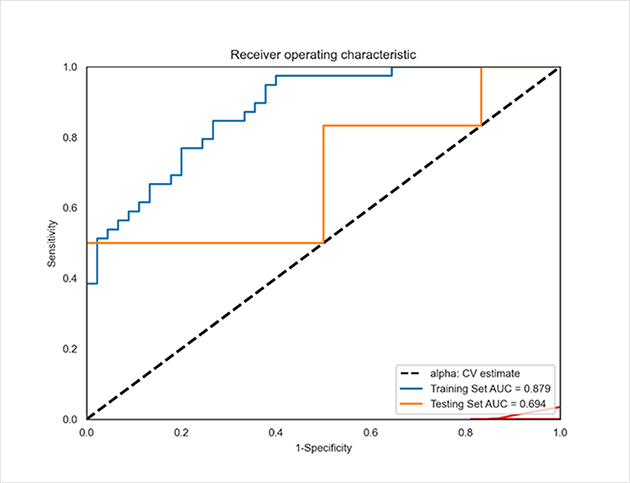


**Supplementary Figure 2.** Receiver operating characteristic curves of radiomics model based on liner-SVM in training set 2 and testing set 2.
